# Supplementary material for: Efficacy of Five Disinfectant Products Commonly Used in Pig Herds against a Panel of Bacteria Sensitive and Resistant to Selected Antimicrobials
Source: Animals (Basel). 2022 Oct 15;12(20):2780. doi: 10.3390/ani12202780 (PMC9597786; doi:10.3390/ani12202780)
Supplement: Supplementary file 1 [file animals-12-02780-s001.zip › Table S1.pdf]

Table S1. Minimum inhibitory concentration (MIC) and minimum bactericidal concentration (MBC) of five disinfectant products against 15 bacterial strains (sorted by disinfectant).

Disinfectant	Bacterial strains	GO	MIC	MBC	MIC/MBC	MBC categories [a]
Disinfectant A	*Enterococcus hirae*	100	200	159	1.26	S
	Staphylococcus aureus	100	6,400	6,041	1.06	ES
	Proteus vulgaris	100	504	94	5.34	T
	Pseudomonas aeruginosa	100	2,263	1,695	1.33	ES
	Salmonella Enteritidis	100	224	53	4.24	T
	S - *E. coli* (4526)	100	283	283	1.00	PS
	S - *E. coli* (4527)	100	200	200	1.00	S
	S - *E. coli* (4529)	100	200	200	1.00	S
	S - *E. coli* (4531)	100	200	200	1.00	S
	S - *E. coli* (25922)	100	200	200	1.00	S
	MDR - *E. coli* (4512)	100	200	26	7.55	T
	MDR - *E. coli* (4534)	100	200	25	8.00	T
	MDR - *E. coli* (4536)	100	200	40	5.04	T
	MDR - *E. coli* (2229)	100	200	26	7.55	T
	MDR - *E. coli* (MSG17 C20)	100	200	53	3.78	T
Disinfectant B	*Enterococcus hirae*	50	1,796	1,131	1.59	ES
	Staphylococcus aureus	50	5,702	673	8.48	ES
	Proteus vulgaris	50	898	141	6.35	PS
	Pseudomonas aeruginosa	50	1,425	283	5.04	ES
	Salmonella Enteritidis	50	566	189	3.00	ES
	S - *E. coli* (4526)	50	504	400	1.26	ES
	S - *E. coli* (4527)	50	400	400	1.00	ES
	S - *E. coli* (4529)	50	504	317	1.59	ES
	S - *E. coli* (4531)	50	400	283	1.41	ES
	S - *E. coli* (25922)	50	504	378	1.33	ES
	MDR - *E. coli* (4512)	50	449	67	6.73	S
	MDR - *E. coli* (4534)	50	283	50	5.66	S
	MDR - *E. coli* (4536)	50	317	100	3.17	PS
	MDR - *E. coli* (2229)	50	400	106	3.78	PS
	MDR - *E. coli* (MSG17 C20)	50	356	168	2.12	ES
Disinfectant C	*Enterococcus hirae*	49	196	156	1.26	ES
	Staphylococcus aureus	49	69	41	1.68	T
	Proteus vulgaris	49	55	35	1.59	T
	Pseudomonas aeruginosa	49	196	156	1.26	ES
	Salmonella Enteritidis	49	98	37	2.67	T
	S - *E. coli* (4526)	49	196	73	2.67	S
	S - *E. coli* (4527)	49	175	98	1.78	S
	S - *E. coli* (4529)	49	175	110	1.59	PS
	S - *E. coli* (4531)	49	196	78	2.52	S
	S - *E. coli* (25922)	49	196	196	1.00	ES
	MDR - *E. coli* (4512)	49	156	37	4.24	T
	MDR - *E. coli* (4534)	49	123	28	4.49	T
	MDR - *E. coli* (4536)	49	156	29	5.34	T
	MDR - *E. coli* (2229)	49	175	28	6.35	T
	MDR - *E. coli* (MSG17 C20)	49	98	29	3.36	T

Table S1. *Cont.*

Disinfectant	Bacterial strains	GO	MIC	MBC	MIC/MBC	MBC categories [a]
Disinfectant D	*Enterococcus hirae*	33	8,448	6,705	1.26	ES
	Staphylococcus aureus	33	135,168	135,168	1.00	ES
	Proteus vulgaris	33	33,792	33,792	1.00	ES
	Pseudomonas aeruginosa	33	67,584	67,584	1.00	ES
	Salmonella Enteritidis	33	22,627	3,364	6.73	ES
	S - *E. coli* (4526)	33	23,895	23,895	1.00	ES
	S - *E. coli* (4527)	33	23,895	23,895	1.00	ES
	S - *E. coli* (4529)	33	21,288	21,288	1.00	ES
	S - *E. coli* (4531)	33	16,896	16,896	1.00	ES
	S - *E. coli* (25922)	33	33,792	33,792	1.00	ES
	MDR - *E. coli* (4512)	33	21,288	16,896	1.26	ES
	MDR - *E. coli* (4534)	33	21,288	18,965	1.12	ES
	MDR - *E. coli* (4536)	33	30,105	8,950	3.36	ES
	MDR - *E. coli* (2229)	33	33,792	26,821	1.26	ES
	MDR - *E. coli* (MSG17 C20)	33	16,000	4,110	3.89	ES
Disinfectant E	*Enterococcus hirae*	360	404	360	1.12	S
	Staphylococcus aureus	360	1,440	720	2.00	PS
	Proteus vulgaris	360	1,814	1,814	1.00	ES
	Pseudomonas aeruginosa	360	360	240	1.50	T
	Salmonella Enteritidis	360	1,000	707	1.41	S
	S - *E. coli* (4526)	360	360	360	1.00	S
	S - *E. coli* (4527)	360	360	360	1.00	S
	S - *E. coli* (4529)	360	360	360	1.00	S
	S - *E. coli* (4531)	360	360	360	1.00	S
	S - *E. coli* (25922)	360	720	454	1.59	S
	MDR - *E. coli* (4512)	360	360	255	1.41	T
	MDR - *E. coli* (4534)	360	509	286	1.78	T
	MDR - *E. coli* (4536)	360	360	255	1.41	T
	MDR - *E. coli* (2229)	360	404	255	1.59	T
	MDR - *E. coli* (MSG17 C20)	360	1,000	707	1.41	S

GO: General Order; MIC: minimum inhibitory concentration; MBC minimum bactericidal concentration; S: sensitive; MDR:. multidrug resistan; MBC score: T (tolerant, MBC>GO); S (sensitive, MBC≤GO); PS (particularly sensitive, MBC≤2×GO); ES (extremely sensitive, MBC≤3×GO).
