# Supplementary material for: Efficacy of Five Disinfectant Products Commonly Used in Pig Herds against a Panel of Bacteria Sensitive and Resistant to Selected Antimicrobials
Source: Animals (Basel). 2022 Oct 15;12(20):2780. doi: 10.3390/ani12202780 (PMC9597786; doi:10.3390/ani12202780)
Supplement: Supplementary file 1 [file animals-12-02780-s001.zip › Table S2.pdf]

Table S2. Minimum inhibitory concentration (MIC) and minimum bactericidal concentration (MBC) of five disinfectant products against 15 bacterial strains (sorted by bacterial strain).

Bacterial strain	Disinfectant A (GO: 100)		Disinfectant B (GO: 50)		Disinfectant C (GO: 49)		Disinfectant D (GO: 33)		Disinfectant E (GO: 360)	
	**MIC**	**MBC**	**MIC**	**MBC**	**MIC**	**MBC**	**MIC**	**MBC**	**MIC**	**MBC**
Salmonella Enteritidis	224	53	566	189	98	37	22,627	3,364	1,000	707
Escherichia coli strain MSG17 C20 (MDR)	200	53	356	168	98	29	16,000	4,110	1,000	707
Escherichia coli strain 4534 (MDR)	200	25	283	50	123	28	21,288	18,965	509	286
Escherichia coli strain 4536 (MDR)	200	40	317	100	156	29	30,105	8,950	360	255
Escherichia coli strain 4512 (MDR)	200	26	449	67	156	37	21,288	16,896	360	255
Escherichia coli strain 2229 (MDR)	200	26	400	106	175	28	33,792	26,821	404	255
Proteus vulgaris	504	94	898	141	55	35	33,792	33,792	1,814	1,814
Staphylococcus aureus	6,400	6,041	5,702	673	69	41	135,168	135,168	1,440	720
Enterococcus hirae	200	159	1,796	1,131	196	156	8,448	6,705	404	360
Pseudomonas aeruginosa	2,263	1,695	1,425	283	196	156	67,584	67,584	360	240
Escherichia coli strain 25922	200	200	504	378	196	196	33,792	33,792	720	454
Escherichia coli strain 4526	283	283	504	400	196	73	23,895	23,895	360	360
Escherichia coli strain 4527	200	200	400	400	175	98	23,895	23,895	360	360
Escherichia coli strain 4529	200	200	504	317	175	110	21,288	21,288	360	360
Escherichia coli strain 4531	200	200	400	283	196	78	16,896	16,896	360	360

GO: recommended in-use concentration; MDR: multidrug resistant; MBC value > GO are highlighted in gray.
